# Professor Caldwell and Animal Chemistry

**Published:** 1844-11

**Authors:** 


PROFESSOR CALDWELL AND ANIMAL CHEMISTRY.

It is not our intention to protract the discussion on animal chemistry by replying to the long communication of our learned correspondent and friend, which is concluded in our present number. We have not been able to find that Professor Caldwell has brought forward in his Reply any material fact or argument not contained in his Critique, which we made the subject of an extended notice, and therefore do not perceive any necessity for an answer to his paper, even if we did not suppose that a disproportionate space in the Journal had already been given to the subject. It were easy to show that his attempt to point out the difference between chemical action and vital action is not successful; and it seems to us that it would not be difficult to prove, that in his comparison of the vital principle to the general of an army, and chemistry to the soldiers, who do everything under the direction of the commander-in-chief, he surrenders the main question between him and Liebig. If we understand Liebig, that is precisely his doctrine. The *vis vitæ*, according to him, presides over and modifies all the functions of living beings in which chemistry is concerned. We have so often quoted Liebig to this effect, that any further reference to his views on that point is deemed quite unnecessary. One more remark is all we shall stop to make concerning the reply of our venerable friend, and that is, that if our notice of his Critique was marked by undue severity, he has meted out the retribution according to his own measure. But we do not complain—we can bear with a great deal from our friends.

